# Population genetic analysis of the *Plasmodium falciparum *6-cys protein Pf38 in Papua New Guinea reveals domain-specific balancing selection

**DOI:** 10.1186/1475-2875-10-126

**Published:** 2011-05-14

**Authors:** John C Reeder, Johanna Wapling, Ivo Mueller, Peter M Siba, Alyssa E Barry

**Affiliations:** 1 Burnet Institute, Melbourne, Australia; 2 Monash University, Melbourne, Australia; 3 Papua New Guinea Institute of Medical Research, Madang, Papua New Guinea

## Abstract

**Background:**

The *Plasmodium falciparum *merozoite surface protein Pf38 is targeted by antibodies of malaria immune adults and has been shown to be under balancing (immune) selection in a Gambian parasite population, indicating potential as a malaria vaccine candidate. This study explores the population genetics of *Pf*38 in Papua New Guinea, to determine the extent and geographic distribution of diversity and to measure selective pressure along the length of the gene.

**Methods:**

Using samples collected during community-based cross-sectional surveys in the Mugil and Wosera regions, the *Pf38 *genes of 59 *P. falciparum *isolates were amplified and sequenced. These sequences, along with previously sequenced Gambian and laboratory isolates, were then subjected to an array of population genetic analyses, examining polymorphisms, haplotype diversity and balancing selection. In addition to whole-gene analysis, the two 6-cys domains were considered separately, to investigate domain specific polymorphism and selection.

**Results:**

Nineteen polymorphic sites were identified in the *Pf *38 gene. Of these, 13 were found in the Gambia, 10 in Mugil and 8 in Wosera. Notably, the majority of common polymorphisms were confined to domain I. Although only moderate levels of nucleotide diversity were observed, the haplotype diversity was high in all populations, suggesting extensive recombination. Analyses of the full-length sequence provided only modest evidence for balancing selection. However, there was a strong contrast between domain I, which showed strong evidence for positive balancing selection, and domain II which was neutral. Analyses of the geographic distribution of Pf38 haplotypes showed that four haplotypes accounted for the majority of sequences found world-wide, but there were many more haplotypes unique to the African than the PNG populations.

**Conclusion:**

This study confirmed previous findings that *Pf38 *is a polymorphic gene under balancing selection. However, analysing polymorphism and selection across the length of the gene painted a considerably different picture. Domain I is highly polymorphic and the target of significant balancing selection. In contrast, domain II is relatively conserved and does not show evidence of immune selective pressure. The findings have implications for future population genetic studies on vaccine candidates, showing that the biological context must also be considered as a framework for analysis.

## Background

For a brief window of time in the asexual lifecycle of *Plasmodium falciparum*, the parasite is exposed to the host immune response as it seeks to engage with and invade an erythrocyte. Intervention at this point, targeting the merozoite to interrupt the mechanism of invasion, is a strongly pursued strategy of malaria vaccine development. Many different parasite proteins have been implicated in the invasion cycle of merozoite attachment, re-orientation and entry [1]. The surface coat of the merozoite is largely comprised of glycophosphatylinositol (GPI) anchored membrane proteins and their associated partners [2]. Nine of these GPI-anchored merozoite surface antigens (MSAs) have been described so far, in raft like membranes [3], and all are potentially erythrocyte ligands. The GPI-anchored proteins MSP-1 and MSP-2 have long been recognized as potential vaccine targets, and they have already been developed and trialled as asexual blood-stage vaccine candidates [4]. Three more GPI-anchored MSAs have more recently come to attention as potential vaccine candidates [5]. These proteins are members of a six-cysteine (Cys_6_) family, whose 11 known members are expressed at various stages of the parasite lifecycle, mostly in the sexual stages [6]. The three asexual stage Cys_6 _proteins, Pf12, Pf38 and Pf41 have been localized to the merozoite surface, with Pf38 distributing more specifically to the apical end than the other two. All three are well-recognized by human immune sera [3], and thus may be involved in the host immune response. However, antibody recognition alone is not substantial proof that an antigen is a sufficiently important target of the protective response to be worthy of prioritization as a vaccine candidate.

Evidence of selective pressure on a particular antigen in a natural parasite population is a good indicator of its importance as a target of human immunity. Using a population genetic approach, this selective pressure can be identified through a signature of balancing selection, detecting the maintenance of low to medium allele frequencies within a parasite population that result in an advantage for immune evasion [7,8]. Recently, a prospective study was undertaken in a Gambian parasite population to identify balancing selection among 26 blood-stage surface-exposed proteins [9], including the Cys_6 _proteins Pf12, Pf38 and Pf92. An initial screen of the antigens by Hudson-Krietman-Aguade (HKA) [10] and McDonald-Krietman (MK) [11] analysis, that calculate divergence from the sequence of *Plasmodium reichenowi *[12], led to the identification of six antigens potentially under selection. Further population-based analysis, using Tajima's D (TjD) test [13] and Fu and Li's indices [14], confirmed four of these (*msp*3/6, *sera*5, *Pf*38 and *msp*7) to have signatures of balancing selection [9].

On the basis of these findings, further exploration of the population genetics of *Pf*38 in two regions of intense perennial malaria transmission in Papua New Guinea was undertaken. The Mugil area in Madang Province, on the north coast of PNG, had been the site of a previous study in 2004, looking at the risk of infection and disease in PNG children attending the local elementary schools [15]. Likewise, the Wosera region of East Sepik Province had been the subject of extensive cross-sectional surveys in 2001-2003 [16]. This previous work provided us with a detailed background of transmission and infection dynamics in these two catchments. In more recent all-age cross-sectional community-based surveys in Mugil and Wosera, infection prevalence (~40%) was found to be similar to the published literature [17]. Genotyping of ten microsatellite loci from these samples uncovered high levels of genetic diversity (allelic richness and heterozygosity) and a lack of linkage disequilibrium [17]. The catchments also had strong local parasite population structure, indicating limited gene flow. The genetic structure of these two populations suggested that this sample set would serve as an excellent indicator of the diversity and selection of *Pf*38 in a malaria endemic area.

## Methods

### Study site and *P. falciparum *isolates

The Papua New Guinean provinces of Madang and East Sepik are areas of intense perennial malaria transmission that have long been the focus of malaria research and ongoing control efforts. As part of a large cross-sectional malaria survey in March 2006, venous blood samples were collected from asymptomatic human volunteers of all ages, including the three villages of Dimer, Karkum and Matukar/Bunu, within the catchment of the Mugil Health Centre, on the North Coast some 50 km from the urban population centre of Madang Town. A similar survey took place in the Wosera region of East Sepik Province in September 2005, including the villages of Gwinyingi and Nindigo. This allowed the examination of a broad range of parasites circulating in the community, unbiased by symptomatic outcomes. Genomic DNA was extracted from the whole blood samples using the 96 well QiaQuick DNA extraction kit (Qiagen). Ethical approval to conduct the study was granted by the PNG Institute of Medical Research Institutional Review Board, the Papua New Guinea Medical Research Advisory Committee and the Alfred Hospital Research and Ethics Unit.

To identify *P. falciparum *positive samples and concurrently estimate prevalence and multiplicity of infection (MOI), samples were screened by genotyping the highly polymorphic gene encoding merozoite antigen protein 2 (*msp2*), as previously described [18]. This approach utilized a nested multiplex PCR to simultaneously amplify both 3D7 and FC27 allele families from genomic DNA. Fluorescently labelled products specific to allele-family were then analysed on an ABI sequencer (Applied Biosystems). As described previously, *P. falciparum *positive samples found to contain a single *msp2 *allele were then screened further at ten microsatellite loci to confirm single infections [17].

### Amplification and sequencing of *Pf38*

Thirty-six single infection gDNA samples from Mugil and twenty-three from the Wosera were amplified for the *Pf*38 gene, using a hemi-nested PCR technique. First round amplification was performed with oligonucleotide primers F2: 5'-ggt caa tca tta cag gaa tcg-3' and R1: 5'-cgc aaa tga aat ttc ttc tc-3' and second round with oligonucleotide primers F2: 5'-ggt caa tca tta cag gaa tcg-3' and R3: 5'-gct tgt taa ctc caa tac ttc-3'. Amplification was performed in 15 μL volumes, containing 1.5 mM MgCl_2_, 200 μM each dNTP, 0.33 μM oligonucleotide primer, 5% PCR grade DMSO (Sigma-Aldrich, MO, USA) and 0.5 units hotstart *Taq *polymerase (5 PRIME, GmbH Germany) The cycling conditions were 94°C for 2 min, 35 cycles of 94°C for 20 sec, 54°C for 30 sec and 60°C for 1 min, followed by a final extension at 60°C for 7 min. Identical protocols for the PCR reaction mix and cycling conditions were used for both rounds of amplification. The resulting 921bp fragment was then purified and sequenced by the commercial sequencing service provided by Macrogen Inc. (Seoul, Korea) on an ABI 3730XL DNA analyser.

### Sequence analysis

Raw sequence data chromatograms were edited and aligned into contigs in Sequencher version 4.0 (Gene Codes). Single nucleotide polymorphisms (SNPs) were identified by comparing each set of sequences for an individual isolate to that of the 3D7 reference genome [19] (Plasmo DB Id: PFE0395c). Individual SNPs were confirmed if they were also identified in other isolates or if detected in at least two independent PCR products from the same isolate. Determination was made of the total number of polymorphic sites (S); the numbers of synonymous (dS) and non-synonymous (dN) polymorphisms; the nucleotide diversity (P), which is the average number of nucleotide differences per site between two sequences [20]; the number of distinct haplotypes (h) and the haplotype (gene) diversity (Hd) which is analogous to heterozygosity (*Hd *= [n/(n-1)][(1-Σ(*f*_*i*_)^2^)] where n is the sample size and *f *is the frequency of the *i*^*t*^^h ^allele) [20]. The number of haplotypes (h) is strongly influenced by sample size therefore we also calculated the allelic richness (*R*_*s*_) which is normalized on the basis of the smallest sample size and based on the rarefaction method developed by Hurlbert [21] and implemented in FSTAT version 2.9.3 software [22]. The nucleotide divergence (K) was measured as the average proportion of nucleotide differences between species [20] using the *P. reichenowi Pf38 *orthologue (GenBank accession: EF123272).

Natural selection was measured using the McDonald-Kreitman test of the neutral hypothesis [11,23], based on a comparison of synonymous and nonsynonymous variation within and between species. Under neutrality, the ratio of replacement to synonymous fixed substitutions (differences) between species should be the same as the ratio of replacement to synonymous polymorphisms within species. Significant increases or decreases in this ratio were determined by two-tailed Fishers exact test. We also calculated the *D *test statistic proposed by Tajima [13], for testing the hypothesis that all mutations are selectively neutral [23]. The D test is based on the differences between the number of segregating sites (S) and the average number of nucleotide differences between sequence pairs. Positive values of Tajima's D indicate balancing selection while negative values indicate purifying or directional selection. A sliding window approach was used to highlight specific regions of *Pf38 *that deviate from neutral expectations. Measurement was made of the F statistic proposed by Fu and Li [14] for testing the hypothesis that all mutations are selectively neutral [23] with the *P.reichenowi Pf38 *sequence as an outgroup; the Hudson, Kreitman and Aguadé's [10] ratio (HKAr) was calculated as P/K and is a relative index used to compare different loci. The test is based on the Neutral Theory of Molecular Evolution [23] prediction that regions of the genome that evolve at high rates will also present high levels of polymorphism within species. All of the above statistics were determined using DnaSP version 5.0 [24]. We also used codon-based Z tests, averaging over all sequences, to calculate the probability of departure from neutrality (d_N _= d_S_), positive selection (d_N _> d_S_) or negative selection (d_N _< d_S_). Values of *p *less than 0.05 were considered significant at the 5% level. The variance of the difference was computed using the bootstrap method (1000 replicates). Analyses were conducted using the Nei-Gojobori method in MEGA4 [20,25]. The interpopulation differentiation (*F*_*ST*_) at specific polymorphic sites was measured using GENEPOP version 4.0 ("GENEPOP on the web") [26].

**Figure 1:**
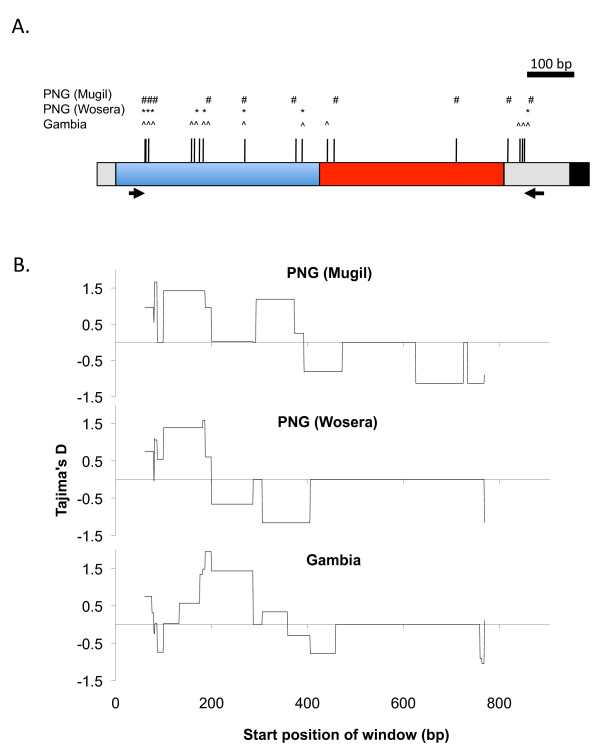
**Pf38 domain structure, polymorphism and signatures of natural selection**. (A) Map of the *Pf38 *gene indicating the positions of primers (arrows), 6-cys domain I (blue) and domain II (red) and GPI anchor (black). Bars indicate positions of SNPs, with symbols above indicating those found in each natural population (B) Sliding window analysis of natural selection (Tajima's D values) for each population. Window size = 100bp, Step size = 3 bp.

As the *Pf38 *gene has two distinct 6-cys domains (Figure 1A) in addition to defining the above statistics for the full sequence, analysis was also performed on the two 6-cys domains separately, to investigate domain specific selective pressure (domain I: 85-466 bp, domain II: 467-892 bp).

**Figure 2:**
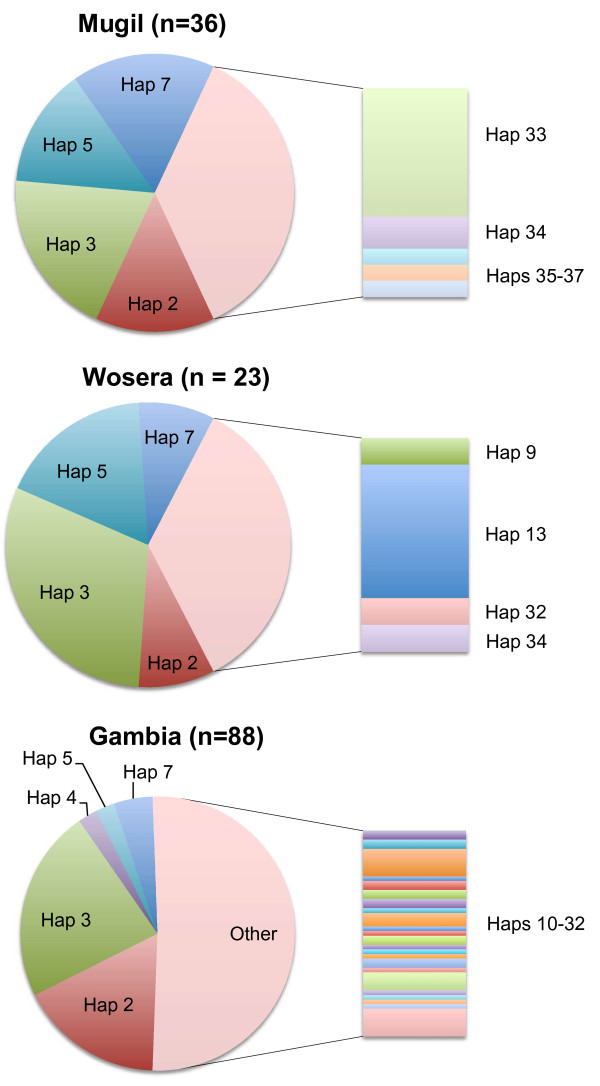
**Geographic distribution of Pf38 amino acid haplotypes**. Pie charts depicting the frequency of haplotypes found in each of the three natural populations.

## Results

### Polymorphism and diversity of *Pf*38

High quality, full-length sequences were obtained for a total of 59 PNG isolates (GeneBank accession#: JF944833-JF944891). These were compared to previously published data from the Gambia (n = 88) and that available for established laboratory isolates (n = 18) [9]. The full dataset is included in the supporting material (Additional File 1). A total of 19 SNP's were identified in the region analysed (85-892 bp). Of these, 13 polymorphic sites were found in the Gambia, 10 were found in Mugil and 8 in Wosera (Figure 1). Polymorphic sites unique to specific populations were also identified, including 5 in the Gambia, 4 in Mugil and 3 in laboratory isolates. No unique sites were present in the Wosera. All of the polymorphisms identified in PNG were non-synonymous mutations, and the only synonymous mutation was found in a single isolate from the Gambia. Although there was a modest level of nucleotide diversity (∏), there were several haplotypes (h) and a high haplotype diversity (Hd) (Table 1). The majority of common polymorphisms were confined to domain I (Figure 1A) while those found in domain II were found at low frequencies being represented in only a few haplotypes (Additional File 2). Accordingly, the ∏ and Hd values for domain I are substantially higher than domain II and much higher than would have been assumed from the whole-gene calculation.

**Table 1:** Analysis of polymorphism and selection in Pf38 sequences (85-892 bp) in different *P. falciparum *populations

										Codon based Z tests (p-value)			McDonald-Kreitman					Allele frequency based tests	
	
	n	**S**^**a**^	dN	dS	h	Hd	**Π (×10**^**3**^)	**K (×10**^**3**^)	HKAr	dN = dS	dN > dS	dN < dS	SYN		NONSYN			Tajima's D	Fu & Li F
													Fixed	Poly	Fixed	Poly	p-value		
**Full **(85-892bp)																			
Mugil	36	10	9	0	11	0.88	3.24	18.97	0.17	0.014*	0.007*	1	4	0	9	9	0.115	0.331	0.303
Wosera	23	8	7	0	9	0.86	2.81	18.24	0.15	0.067	0.035*	1	4	0	9	7	0.248	0.151	-0.397
Gambia	88	13	11	1	36	0.95	4.04	19.09	0.21	0.015*	0.007*	1	4	1	9	11	0.321	0.432	1.057
**Domain I **(85-466bp)																			
Mugil	36	6	6	0	6	0.82	5.74	18.33	0.31	0.023*	0.012*	1	4	0	1	6	0.015*	1.407	1.475^#^
Wosera	23	7	7	0	8	0.85	5.71	17.53	0.33	0.026*	0.012*	1	4	0	1	7	0.010*	0.475	0.108
Gambia	88	9	8	1	23	0.90	6.69	17.85	0.37	0.021*	0.009*	1	4	1	1	8	0.0229*	1.106	0.908
**Domain II **(467-892bp)																			
Mugil	36	4	3	0	5	0.35	0.99	19.55	0.05	0.101	0.052	1	0	0	8	3	n/a	-1.3773	-1.336
Wosera	23	1	n/a	n/a	2	0.09	0.20	18.88	0.01	0.295	1	0.151	0	0	8	0	n/a	-1.161	-1.640
Gambia	88	4	3	0	4	0.60	1.63	20.20	0.08	0.32	0.154	1	0	0	8	3	n/a	-0.247	0.668

*significant (p < 0.05) #aproaching significance (p < 0.10) n/a = not applicable

n = number of sequences, S = number of polymorphic sites, dN = nonsynonymous polymorphisms, dS = synonymous polymorphisms, h = haplotypes, Hd = Haplotype diversity, Π = nucleotide diversity, K = interspecific divergence, HKAr = HK, A ratio (Π/K)

^a^There is a polymorphism within an incomplete codon at the 3' end of the sequence, preventing determination of NS or Syn, which explains the lower number of dN+dS than S

### Signatures of balancing selection in domain I of *Pf*38

Signatures of balancing selection are strong indicators of immune selection and a number of the analyses in Table 1 address this question (HKAr, Z test, M-K, TjD and Fu & Li F). All of these tests showed the same trend, with the full gene demonstrating limited evidence of balancing selection. However, there was a strong contrast observed between domain I, with significant departure from neutrality in a number of tests, and domain II, which showed neutral values. The specific location of the selective pressure on the gene is clearly illustrated by the Tajima's D plot in Figure 1B, where positive values represent balancing selection and negative values represent purifying or directional selection. Low levels of interpopulation differentiation (*F*_ST_) for several sites in domain I further support the activity of balancing selection on this region compared to domain II where higher levels of *F*_ST _were observed (though small sample sizes may influence these results).

### Geographic distribution of *Pf*38 haplotypes

A total of 37 different *Pf*38 haplotypes encoding distinct amino acid sequences were observed among the combination of the PNG sequences generated in the current study and the Gambian and laboratory isolates from previous investigations [9] (Table 2 and Additional File 3). The Gambian Pf38 sequences showed the greatest diversity with 28 of the total 37 haplotypes represented. This increased diversity was also observed when the allelic richness (*R*_S_), which provides a normalized value on the basis of the smallest sample size (n = 23), was measured (*R*_S_: Gambia = 18.449, Mugil = 8.604 and Wosera = 8.00). Three haplotypes were unique to laboratory isolates and not observed in the natural populations analysed. The sequences from the Wosera and Mugil contained far fewer haplotypes, with 8 and 9 respectively, which will at least in part be the result of a smaller sample size than the Gambia. Interestingly, only four haplotypes (Haps 2,3,5 and 7) were found to account for over 60% of the PNG isolates and almost half of those from the Gambia. In the sector outside these common globally distributed haplotypes there is considerable diversity, not only between Africa and PNG, but also between the two PNG locations. In Mugil, a single polymorphism seen at position 416 and found in Haps 33,35,36 and 37 is present in 30% of samples, but is not seen in either the Wosera or Gambian samples. Likewise in Wosera, a polymorphism at position 205 and found in Hap13 is seen in 20% of samples, yet not seen in the Wosera and only found in one isolate from the Gambia.

## Discussion

The GPI-anchored *P. falciparum *merozoite membrane protein Pf38 is a potentially attractive candidate for vaccine development studies. It is expressed in both sexual and asexual stages and its isolation from raft-like membranes and localization to the merozoite apical complex in the erythrocytic cycle suggests a role as a ligand in erythrocyte invasion [3]. The protein is strongly targeted by the antibodies in the serum of immune PNG adults [3] and 20-mer peptides based on *Pf*38 sequence have been shown to inhibit *in vitro *invasion [5]. In Gambian isolates, *Pf*38 was one of only 4 out of 26 genes encoding surface-exposed proteins that carried a signature of balancing selection, though this was a relatively moderate effect compared to better known vaccine candidates, such as *ama1 *[9]. Study of the population genetics of *Pf38 *from a different geographical location was needed to confirm its susceptibility to immune selection and to explore the degree of diversity and geographical variation within the gene, in order to further consider of its potential as a malaria vaccine candidate.

A substantial number (n = 19) of SNPs were found amongst the isolates. The majority of these were confined to domain I and those appearing in domain II were either singletons or found at extremely low frequencies within the parasite populations. In all three populations, the overall nucleotide diversity (∏) was low, yet the haplotype diversity (Hd) was high. This has been previously observed in analysis of other merozoite surface antigen genes, such as *eba175*, and suggests that recombination generates a range of haplotypes even where functional constraints exist [17]. When considering domain I alone, nucleotide diversity is much higher than domain II and substantially higher than the figure for the full gene. This might suggest some functional limitation of the amount of polymorphism that can be maintained in domain II.

Signatures of balancing selection, demonstrating the maintenance of putative antigenic diversity within populations, have proven a good indication of the importance of antigens as targets of the immune response [7,8]. It is important to consider a range of statistical tests of selection, such as those performed here, as no single test gives definitive proof, and indeed correlation of some of the tests can be quite poor in certain circumstances [9,12]. The various methods utilized here consistently pointed towards a modest degree of balancing selection for the *Pf38 *gene in the PNG population when the full sequences were analysed, as had been seen previously in the Gambian isolates [9]. Of particular interest was the very considerable increase in evidence for balancing selection in domain I, compared to the full gene and domain II. The sliding window analysis of Tajima's D, with domain I demonstrating strong balancing selection and domain II showing neutral values, combined with the SNP map and observation that the majority of SNPs in domain I were common while those in domain II were rare, suggests that domain I may be the dominant target of natural host immunity.

The greater diversity seen in the Gambian isolates is confirmed by the presence of many more unique haplotypes than that observed in the PNG populations. Of the 34 haplotypes seen in field isolates, 28 are represented in the Gambian dataset. Wosera and Mugil display 8 and 9 haplotypes respectively. Of importance, in the context of the possibility of creating a vaccine with broad coverage of the worldwide parasite population, is that the majority of isolates in each natural population harboured one of just four dominant haplotypes. Aside from these common haplotypes, there is quite considerable variation, with several unique haplotypes in each population.

Taking this information as a whole, one could speculate that the antigen has a functionally restricted domain II shielded from the immune response by an exposed and highly polymorphic domain I. The hypothesis of functional conservation of domain II is given support by work from Garcia *et al *[5], who performed a fine mapping study of the binding of *Pf38*. They created a set of 20-residue-long peptides covering the complete gene sequence and performed a series of binding experiments, and erythrocyte invasion inhibition assays. Two peptides (aa141-160 and aa201-220) showed high activity binding and binding inhibition. Both of these were located inside domain II, suggesting that this region has a role in protein-protein interactions. None of the SNPs identified in the present study co-localized with these peptides.

## Conclusions

This study of polymorphism, diversity and selection of Pf38 in two *P. falciparum *populations in PNG, confirmed the findings of Tetteh *et al*. [9] in the Gambia that *Pf38 *is a modestly polymorphic antigen gene that shows some evidence of balancing selection. However, the analysis of population data in a domain specific fashion here has painted a considerably different picture of the gene than that assumed from the full-length analysis. Domain I is highly polymorphic and shows evidence of significant balancing selection, suggesting that the gene is a target of natural immunity. Domain II is relatively conserved with no evidence of immune selection, supporting the hypothesis that it may be the functionally-conserved site of protein-protein interaction in the invasion process. The findings here have implications for future population genetic studies on vaccine candidates. They demonstrate that direct whole-gene comparisons may give misleading rankings of vaccine potential. One must instead consider the structural and functional context as a framework for analysis.

## Competing interests

The authors declare that they have no competing interests.

## Authors' contributions

JCR performed experiments and analysis and drafted the paper. JW performed experiments and helped write the paper. IM co-ordinated the field studies, provided samples and helped interpret the results. PMS provided logistical support. AEB performed data analysis and helped write the paper. All authors read and approved the final manuscript.

## Supplementary Material

Additional file 1 **Sequence dataset**. FastA formatted file of all Pf38 sequences used in the analysis, including those from Gambian, PNG and laboratory isolates. Click here for file

Additional file 2 **Single nucleotide polymorphisms in Pf38**. Table of single nucleotide polymorphisms found in *Pf38 *sequences from PNG isolates relative to the reference sequence (from clone 3D7). The table indicates the presence of nucleic acid changes in PNG isolates against the total 19 SNPS identified in PNG, Gambian and laboratory isolates. Click here for file

Additional file 3 **Amino acid haplotypes for Pf38**. Table of amino acid haplotypes found in *Pf38 *from PNG the Gambia and laboratory isolates. The frequency of their occurrence in each population is indicated on the right and the laboratory isolates are listed against their haplotypes. Click here for file
