# Supplementary material for: Population genetic analysis of the Plasmodium falciparum 6-cys protein Pf38 in Papua New Guinea reveals domain-specific balancing selection
Source: Malar J. 2011 May 14;10:126. doi: 10.1186/1475-2875-10-126 (PMC3112457; doi:10.1186/1475-2875-10-126)
Supplement: Additional file 2 — Single nucleotide polymorphisms in Pf38. Table of single nucleotide polymorphisms found in Pf38 sequences from PNG isolates relative to the reference sequence (from clone 3D7). The table indicates the presence of nucleic acid changes in PNG isolates against the total 19 SNPS identified in PNG, Gambian and laboratory isolates. [file 1475-2875-10-126-S2.PDF]

	Locus	103_104		110	199	205	215	223	310	416	429	482	496	553	599	749	857	883	887	892*
	3D7 codon (residue)	gga (G)		ttc (F)	gaa (E)	gaa (E)	gac (D)	aaa (K)	gaa (E)	aag (K)	aga (R)	gct (A)	tcg (S)	gta (V)	gat (E)	atg (M)	aat (N)	aac (N)	aca (T)	t
	Variant codon (residue)	aga(R) or aaa (K)		tcc (S)	aaa (K)	aaa (K)	gtc (V)	caa (Q)	aaa (K)	agg (R)	aga (S)	gtt (V)	ccg (P)	ata (I)	ggt (V)	acg (T)	agt (S)	tac (Y)	ata (I)	c
MUGIL	MUG3	:	:	:	:	:	:	C	A	:	:	:	:	:	:	:	:	:	:	C
	MUG16	:	:	:	:	:	:	C	A	:	:	:	:	:	:	:	:	:	:	C
	MUG17	:	:	:	:	:	:	:	:	:	:	:	:	:	:	:	:	:	:	C
	MUG18	:	:	:	:	:	:	:	:	:	:	:	:	:	:	:	:	:	:	:
	MUG22	A	:	C	:	:	:	:	:	G	:	:	:	:	:	:	:	:	:	C
	MUG23	A	A	:	:	:	:	C	:	:	:	:	:	:	:	:	:	:	:	C
	MUG25	:	:	:	:	:	:	:	:	:	:	:	:	:	:	:	:	:	:	C
	MUG26	A	:	:	:	:	:	C	:	:	:	:	:	:	:	:	:	:	:	C
	MUG27	A	:	:	:	:	:	C	:	:	:	:	:	:	:	:	:	N/A	N/A	N/A
	MUG31	A	:	C	:	:	:	:	:	:	:	:	:	:	:	:	:	:	:	:
	MUG32	A	A	:	:	:	:	C	:	:	:	:	:	:	:	:	:	:	:	C
	MUG33	A	:	C	:	:	:	:	:	G	:	:	:	:	:	:	:	:	:	C
	MUG36	A	:	:	:	:	:	C	:	:	:	:	:	:	:	:	:	:	:	C
	MUG37	A	:	C	:	:	:	:	:	:	:	:	:	:	:	:	:	:	:	C
	MUG41	A	:	C	:	:	:	:	:	:	:	:	:	:	:	:	:	:	:	C
	MUG42	:	:	:	:	:	:	C	A	:	:	:	:	:	:	:	:	:	:	C
	MUG51	A	:	C	:	:	:	:	:	:	:	:	:	:	:	:	:	:	:	C
	MUG58	A	:	C	:	:	:	:	:	:	:	:	:	:	:	:	:	:	:	C
	MUG59	A	:	C	:	:	:	:	:	G	:	:	:	:	:	:	:	:	:	C
	MUG62	A	:	C	:	:	:	:	:	:	:	:	:	:	:	:	:	:	:	C
	MUG64	:	:	:	:	:	:	:	:	:	:	:	:	:	:	:	:	:	:	C
	MUG65	A	:	C	:	:	:	:	:	G	:	:	:	:	:	:	:	:	:	C
	MUG66	A	:	:	:	:	:	C	:	:	:	:	:	:	:	:	:	:	:	C
	MUG68	A	:	C	:	:	:	C	A	:	:	:	:	:	:	:	:	:	:	C
	MUG71	A	:	C	:	:	:	:	:	G	:	:	:	:	:	:	:	:	:	C
	MUG73	A	:	C	:	:	:	:	:	G	:	:	:	:	:	:	:	:	:	C
	MUG77	:	:	:	:	:	:	:	:	:	:	:	:	:	:	:	:	:	:	:
	MUG82	A	:	C	:	:	:	:	:	G	:	:	:	:	:	:	:	:	:	C
	MUG85	A	:	C	:	:	:	:	:	G	:	:	C	:	:	:	:	:	:	C
	MUG86	A	:	:	:	:	:	C	:	:	:	:	:	:	:	:	:	:	:	C
	MUG89	A	:	C	:	:	:	:	:	G	:	:	:	:	:	:	:	:	:	C
	MUG90	A	:	:	:	:	:	C	:	:	:	:	:	:	:	:	:	:	:	C
	MUG95	A	:	C	:	:	:	:	:	G	:	:	:	:	:	C	:	:	:	C
	MUG97	A	:	:	:	:	:	C	:	:	:	:	:	:	:	:	:	:	:	C
	MUG98	A	:	C	:	:	:	:	:	G	:	:	C	:	:	:	G	:	:	C
	MUG99	:	:	:	:	:	:	:	:	:	:	:	:	:	:	:	:	:	:	:
	MUG102	:	:	:	:	:	:	C	A	:	:	:	:	:	:	:	:	:	:	C
WOSERA	GWIN7	:	:	:	:	A	:	C	:	:	:	:	:	:	:	:	:	:	:	C
	GWIN8	:	:	:	:	A	:	C	:	:	:	:	:	:	:	:	:	:	:	C
	GWIN9	:	:	:	:	:	:	:	:	:	:	:	:	:	:	:	:	:	:	:
	GWIN11	A	:	C	:	:	:	:	:	:	:	:	:	:	:	:	:	:	:	C
	GWIN12	A	:	:	:	:	:	C	:	:	C	:	:	:	:	:	:	:	:	C
	GWIN15	A	:	C	:	:	:	:	:	:	:	:	:	:	:	:	:	:	:	C
	GWIN17	A	:	C	:	:	:	:	:	:	:	:	:	:	:	:	:	:	:	C
	GWIN24	:	:	:	:	A	:	C	:	:	:	:	:	:	:	:	:	:	:	C
	NIN3	A	:	:	:	:	:	:	:	:	:	:	:	:	:	:	:	:	:	C
	NIN11	A	:	C	:	:	:	:	:	:	:	:	:	:	:	:	:	:	:	C
	NIN12	:	:	:	:	:	:	:	:	:	:	:	:	:	:	:	:	:	:	C
	NIN13	:	:	:	:	:	:	C	A	:	:	:	:	:	:	:	:	:	:	C
	NIN18	A	:	C	:	:	:	:	:	:	:	:	:	:	:	:	:	:	:	C
	NIN23	:	:	:	:	A	:	C	:	:	:	:	:	:	:	:	:	:	:	C
	NIN25	:	:	C	:	:	:	C	A	:	:	:	:	:	:	:	:	:	:	C
	NIN27	A	A	:	:	:	:	C	:	:	:	:	:	:	:	:	:	:	:	C
	NIN33	:	:	:	:	:	:	:	:	:	:	:	:	:	:	:	:	:	:	C
	NIN34	:	:	:	:	:	:	:	:	:	:	:	:	:	:	:	:	:	:	C
	NIN37	A	:	:	:	:	:	C	:	:	:	:	:	:	:	:	:	:	:	C
	NIN45	A	:	:	:	:	:	C	:	:	:	:	:	:	:	:	:	:	:	C
	NIN48	:	:	:	:	A	:	C	:	:	:	:	:	:	:	:	:	:	:	C
	NIN50	A	:	C	:	:	:	:	:	:	:	:	:	:	:	:	:	:	:	C
	NIN52	A	:	C	:	:	:	:	:	:	:	:	:	:	:	:	:	:	:	C

*first position in codon, end of sequenced region
