# Supplementary material for: Population genetic analysis of the Plasmodium falciparum 6-cys protein Pf38 in Papua New Guinea reveals domain-specific balancing selection
Source: Malar J. 2011 May 14;10:126. doi: 10.1186/1475-2875-10-126 (PMC3112457; doi:10.1186/1475-2875-10-126)
Supplement: Additional file 3 — Amino acid haplotypes for Pf38. Table of amino acid haplotypes found in Pf38 from PNG the Gambia and laboratory isolates. The frequency of their occurrence in each population is indicated on the right and the laboratory isolates are listed against their haplotypes. [file 1475-2875-10-126-S3.PDF]

Locus	103_104	110	199	205	215	223	310	416	429	482	496	553	599	749	857	883	887	Wosera	Mugil	Gambia	Laboratory
Hap 1	R	F	E	E	D	Q	E	K	R	A	S	I	G	M	N	N	T				T9/96
Hap 2	G	F	E	E	D	Q	K	K	R	A	S	V	D	M	N	N	T	2	5	15	Dd2,W2mef, FCR3, T9/94
Hap 3	R	S	E	E	D	K	E	K	R	A	S	V	D	M	N	N	T	7	7	20	T9/102, RO33, HB3
Hap 4	R	F	K	E	D	Q	E	K	S	A	S	V	D	M	N	N	T			2	D6
Hap 5	G	F	E	E	D	K	E	K	R	A	S	V	D	M	N	N	T	4	5	2	K1, 3D7
Hap 6	R	F	E	E	V	Q	E	K	R	A	S	V	D	M	N	N	T				Palo Alto, CAMP'
Hap 7	R	F	E	E	D	Q	E	K	R	A	S	V	D	M	N	N	T	2	6	4	D10, MAD20
Hap 8	G	F	E	E	D	K	K	K	R	A	S	V	D	M	N	N	T				7g8
Hap 9	R	F	E	E	D	K	E	K	R	A	S	V	D	M	N	N	T	1			Wellcome, FCC2
Hap 10	R	F	E	E	D	Q	E	K	R	A	S	V	D	M	N	N	T			2	
Hap 11	R	F	E	E	D	Q	E	K	R	A	S	V	D	M	N	N	I			2	
Hap 12	G	F	E	E	D	Q	E	K	R	A	S	V	D	M	N	N	T			6	
Hap 13	G	F	E	K	D	Q	E	K	R	A	S	V	D	M	N	N	T	5		1	
Hap 14	R	S	E	K	D	Q	E	K	R	A	S	V	D	M	N	N	T			2	
Hap 15	R	F	E	E	D	Q	K	K	R	A	S	V	D	M	N	N	T			2	
Hap 16	K	F	E	E	D	Q	K	K	R	A	S	V	D	M	N	N	T			2	
Hap 17	K	F	E	E	D	Q	K	K	S	A	S	V	D	M	N	N	T			1	
Hap 18	K	F	E	K	D	Q	E	K	R	A	S	V	D	M	N	N	T			3	
Hap 19	G	F	E	E	D	K	E	K	R	A	S	V	D	M	N	N	I			1	
Hap 20	K	F	E	K	D	Q	E	K	R	A	S	V	D	M	N	N	T			1	
Hap 21	R	F	E	K	D	Q	E	K	R	V	S	V	D	M	N	N	T			2	
Hap 22	K	F	E	E	D	Q	E	K	R	A	S	V	D	M	N	N	I			1	
Hap 23	R	F	E	K	D	Q	E	K	S	A	S	V	D	M	N	N	T			1	
Hap 24	R	F	E	K	D	Q	E	K	S	A	S	V	D	M	N	Y	T			1	
Hap 25	R	S	E	E	D	Q	E	K	R	A	S	V	D	M	N	N	T			2	
Hap 26	G	F	E	E	D	Q	K	K	R	V	S	V	D	M	N	N	T			1	
Hap 27	R	S	E	E	D	K	K	K	R	A	S	V	D	M	N	N	T			4	
Hap 28	R	S	E	E	D	Q	K	K	R	A	S	V	D	M	N	N	T			1	
Hap 29	G	F	E	E	D	Q	K	K	S	A	S	V	D	M	N	N	T			1	
Hap 30	R	F	E	E	D	K	E	K	S	A	S	V	D	M	N	N	T			1	
Hap 31	K	F	E	E	D	K	K	K	R	A	S	V	D	M	N	N	T			1	
Hap 32	R	F	E	E	D	Q	E	K	S	A	S	V	D	M	N	N	T	1		6	
Hap 33	R	S	E	E	D	K	E	R	R	A	S	V	D	M	N	N	T		8		
Hap 34	K	F	E	E	D	Q	E	K	R	A	S	V	D	M	N	N	T	1	2		
Hap 35	R	S	E	E	D	K	E	R	R	A	S	V	D	T	N	N	T		1		
Hap 36	R	S	E	E	D	K	E	R	R	A	P	V	D	M	N	N	T		1		
Hap 37	R	S	E	E	D	K	E	R	R	A	P	V	D	M	S	N	T		1		
